# Comprehensive Insights into Potential Metabolic Functions of *Myxococcota* in Activated Sludge Systems

**DOI:** 10.1264/jsme2.ME24068

**Published:** 2024-12-27

**Authors:** Hazuki Kurashita, Masashi Hatamoto, Shun Tomita, Takashi Yamaguchi, Takashi Narihiro, Kyohei Kuroda

**Affiliations:** 1 Bioproduction Research Institute, National Institute of Advanced Industrial Science and Technology (AIST), 2–17–2–1 Tsukisamu-Higashi, Toyohira-ku, Sapporo, Hokkaido, 062–8517 Japan; 2 Department of Science of Technology Innovation, Nagaoka University of Technology, 1603–1 Kamitomioka, Nagaoka, Niigata, 940–2188 Japan; 3 Department of Civil and Environmental Engineering, Nagaoka University of Technology, 1603–1 Kamitomioka, Nagaoka, Niigata, 940–2188 Japan

**Keywords:** Myxobacteria, shotgun metagenomic sequence ana­lysis, social behavior, wastewater treatment plants, secondary metabolism synthetic gene clusters

## Abstract

Myxobacteria, belonging to the phylum *Myxococcota*, are ubiquitous in soil, marine, and other environments. A recent metagenomic sequencing ana­lysis showed that *Myxococcota* are predominant in activated sludge systems; however, their metabolic traits remain unclear. In the present study, we exami­ned the potential biological functions of 46 metagenomic bins of *Myxococcota* reconstructed from activated sludge samples from four municipal sewage treatment plants. The results obtained showed that most *Myxococcota* bins had an almost complete set of genes associated with glycolysis and the TCA cycle. The Palsa-1104 and *Polyangiales* bins contained the glycoside hydrolase GH5 and peptidase M23, which are presumably involved in lysis of the cell wall and cellular cytoplasm, suggesting that some *Myxococcota* from activated sludge prey on other microorganisms. The cell contact-dependent predatory functions of *Myxococcus xanthus* are conserved in the family *Myxococcaceae*, but not in other families. Two bins belonging to Palsa-1104 had phototrophic gene clusters, indicating the potential for heterotrophic and autotrophic metabolism by these microbes. In assessments of the social behavior of *Myxococcota* in activated sludge, the FruA gene and C-signal gene, which are involved in the regulation of fruiting body formation, were lacking in *Myxococcota* bins, suggesting their inability to form fruiting bodies. In addition, multiple bins of *Myxococcota* had novel secondary metabolite biosynthesis gene clusters that may be used for the predation of other bacteria in activated sludge. Our metagenome-based ana­lyses provide novel insights into the microbial interactions associated with *Myxococcota* in activated sludge ecosystems.

Myxobacteria, which are ubiquitous in soil and oceans, were first described in 1892 (Thaxter, 1892). Myxobacteria belong to the order *Myxococcales* of the class *Deltaproteobacteria*; however, the recent reclassification of the phylum *Proteobacteria* has led to the proposal of the phyla *Myxococcota*, *Desulfobacterota*, and SAR324 (Langwig *et al.*, 2022; Waite *et al.*, 2020). *Myxococcus xanthus*, the most studied myxobacteria, has a unique life cycle in which myxobacterial cells aggregate and form fruiting bodies upon sensing starvation (referred to as social behavior) and produce spores that are resistant to desiccation (Gokhale, 1947; Koch and White, 1998). Additionally, its genome size (up to 15‍ ‍Mb) (Schneiker *et al.*, 2007; Han *et al.*, 2013) is larger than those of other bacteria and it has various biosynthetic gene clusters (BGCs) for secondary metabolites, such as myxovirescin and myxalamid (Weissman and Müller, 2010).

The formation of fruiting bodies has been confirmed in *M. xanthus*, *Minicystis rosea*, and *Stigmatella aurantiaca* (Silakowski *et al.*, 1996; Garcia *et al.*, 2014). Thirteen modules (enhancer-binding protein (EBP), MrpC, Nla24, exopolysaccharide (EPS) production, FruA, C-signal, A-signal, aggregation-sporulation-fruiting body formation, development timer, adventurous motility, social motility, outer membrane exchange (OME), and chemosensory pathways/rippling) have been exami­ned in detail because they are the gene modules involved in the social behavior of *M. xanthus* (Murphy *et al.*, 2021). In contrast, metagenome-assembled genomes of *Myxococcota* (such as JAFGVO01, JAFGXQ01, and JAFGIB01) obtained from anaerobic sediments of Zodletone hot springs were found to lack genes belonging to the FruA module, C-signal, and EPS production, to the genera *Anaeromyxobacter* and *Labilithrix* (Thomas *et al.*, 2008; Yamamoto *et al.*, 2014; Murphy *et al.*, 2021). Zodletone *Myxococcota* and *Anaeromyxobacter* lacked homologs of sporulation genes (Murphy *et al.*, 2021) and the phenotypic characteristics of *Labilithrix luteola* and *Vulgatibacter incomptus* showed no aggregates, fruiting bodies, or spore formation in pure cultures (Yamamoto *et al.*, 2014). Therefore, the life cycles of the phylum *Myxococcota* vary according to their phylogeny and ecophysiology.

*Myxococcota* are bacterial predators cultivated using macromole­cular substrates, such as *Escherichia coli* and cellulose (Han *et al.*, 2013; Livingstone *et al.*, 2017). A genomic ana­lysis indicated that it encodes multiple peptidases that degrade cell walls and carbohydrate-active enzymes (CAZymes) (Thiery and Kaimer, 2020). In addition, recent studies elucidated the cell-contact predatory function of *M. xanthus*, confirming the importance of the tight adherence (Tad) and Type III secretion systems (T3SS) for both the induction of cell death and lysis (Wang *et al.*, 2023). Isolates and metagenome-assembled genomes of *Myxococcota* from anaerobic environments have been reported to contain genes that are relevant to anaerobic ATP production (such as nitrogen fixation, HydABC, and the Wood-Ljungdahl pathway) and sulfur metabolism (such as QmoABC, AprAB, and DsrAB), which are not present in known aerobic myxobacterial genomes, suggesting that *Myxococcota* have acquired several functional genes for adaptation to different environments (Murphy *et al.*, 2021). Since the metabolic functions of *Myxococcota* in different environments are diverse, the further accumulation of physiological and genomic information through cultivation and genomic ana­lyses is required.

The activated sludge process has been widely used as a biological treatment system for municipal and industrial wastewater. In this process, phylogenetically diverse microorganisms (*e.g.*, bacteria, archaea, and various eukaryotes, such as protozoa) are involved in wastewater remediation, and predatory interactions have been observed between them (Burian *et al.*, 2022; Kuroda *et al.*, 2023). Members of the bacterial phyla *Myxococcota* and *Bdellovibrionota* are well known as predatory microorganisms in the activated sludge process. Recent 16S rRNA-based stable isotope probing (SIP) ana­lyses revealed the active predatory capacity of the phyla *Myxococcota* and *Bdellovibrionota* in activated sludge systems, particularly *Haliangium* and the uncultured mle1-27 clade, which exhibited high predatory activity in activated sludge (Zhang *et al.*, 2023). In our recent study, we successfully isolated *Corallococcus caeni* belonging to the family *Myxococcaceae*, which includes the genus *Myxococcus*, from activated sludge using a traditional culture technique with *E. coli* as a prey bacterium (Tomita *et al.*, 2024); however, we have yet to isolate other myxobacteria, including *Haliangium* and mle1-27, which exhibit high predatory activity in the process (Zhang *et al.*, 2023). Therefore, culture-independent approaches are required to obtain a more detailed understanding of the potential ecological roles of *Myxococcota* in activated sludge. Our previous metagenomic study on 600 activated sludge samples revealed that predatory or parasitic bacteria belonging to *Myxococcota* and *Bdellovibrionota* negatively correlated with total carbon and nitrogen and were predominantly present as core microbes, implying that predatory bacteria that utilize the cellular biomass as a nutrient become predominant in activated sludge systems due to decreases in available carbon and nitrogen components in wastewater. Therefore, predatory bacteria may be relevant to the maintenance of good water quality, such as total carbon and nitrogen removal derived from cell components (Kuroda *et al.*, 2023). A metagenomic study on activated sludge systems showed that *Myxococcota* with large genomes encoded BGCs (Liu *et al.*, 2021). In addition, *Myxococcota* bacteria have the potential to produce various bioactive substances that are beneficial for human health, such as antibiotics (*e.g.*, myxovirescin, myxochromide, and myxalamide) (Korp *et al.*, 2016; Bhat *et al.*, 2021). These findings suggest that *Myxococcota* in activated sludge systems are important microorganisms for stabilizing wastewater treatment processes through predation and the production of BGCs in complex environments. Although the specific functions of activated sludge *Myxococcota*, such as their potential to produce BGCs, predatory activity, and beneficial effects on water quality, have been exami­ned, their comprehensive genomic characteristics remain unclear. The social behavior and metabolic functions (*e.g.*, carbon/nitrogen/sulfur utilization, the potential to produce BGCs, and the presence of predatory-related genes) of *Myxococcota* in the activated sludge process remain largely unknown. A more detailed understanding of the potential metabolic functions of uncovered microorganisms may be useful for elucidating the fate of wastewater pollutants in activated sludge systems and the development of stable treatment biotechnology. To analyze the metabolic potential of *Myxococcota* in the present study, shotgun metagenomic sequencing-based metabolic reconstruction was performed. We herein analyzed 46 metagenomic bins of the phylum *Myxococcota* recovered from four activated sludge processes to elucidate metabolic and ecological functions related to predation as well as wastewater remediation.

## Materials and Methods

### Sampling, DNA extraction, PCR amplification, and a 16S rRNA gene sequence ana­lysis

Activated sludge samples used to treat municipal wastewater were collected from four wastewater treatment plants (WWTPs; E1, F1, G1, and H1) in Japan. These WWTPs have implemented the operation of conventional activated sludge processes. DNA was extracted using a Fast DNA Spin Kit for Soil (MP Biomedicals) according to the manufacturer’s protocol. DNA concentrations were measured using the Qubit dsDNA BR Assay Kit (QIAGEN). PCR amplification of the 16S rRNA gene was performed using a universal forward primer (Univ515F: 5′-GTGCCAGCMGCCGCGGGTAA-3′) and reverse primer (Univ909R: 5′-CCCCGYCAATTCMTTTRAGT-3′) (Kozich *et al.*, 2013) for F1, G1, and H1 and Univ806R: 5′-GGACTACHVGGGTHTCTAAT-3′ for E1 (Caporaso *et al.*, 2012). PCR products were purified using a QIAquick PCR Purification Kit (Qiagen) according to the manufacturer’s protocol. Purified 16S rRNA gene sequences were analyzed using MiSeq Reagent Kit v3 and a MiSeq system (Illumina). Raw 16S rRNA gene sequences were analyzed using QIIME 2 (ver. 2021.4) according to a previous study (Kuroda *et al.*, 2023). 16S rRNA gene sequences were clustered by ≥97% similarity to operational taxonomic units (OTUs) using vsearch software R (Rognes *et al.*, 2016). Taxonomic assignment was performed using classify-sklearn on SILVA database version 138.

### Shotgun metagenomic sequence ana­lysis

The shotgun metagenomic sequencing of DNA extracted from activated sludge samples at the four WWTPs (E1, F1, G1, and H1) was performed using NovaSeq6000 (150 bp ×2). Metagenomic ana­lyses of F1, G1, and H1 were conducted as previously described (Kuroda *et al.*, 2022). Briefly, raw sequence data from each single extracted DNA sample of F1, G1, and H1 were quality trimmed using Trimmomatic 0.39 (Bolger *et al.*, 2014) and assembled using Megahit2 (Li *et al.*, 2015). Metagenome-assembled bins were generated using Das Tool 1.1.2 (Sieber *et al.*, 2018) and binning software from Metabat2 (Kang *et al.*, 2019), MaxBin2 (Wu *et al.*, 2016), and MyCC (MyCC_2017. ova) (Lin and Liao, 2016). Reconstructed bins were assessed for completeness and contamination using CheckM 1.0.7 (Parks *et al.*, 2015). In the present study, we selected bins (belonging to the phylum *Myxococcota*) with ≥80% completeness and ≤10% contamination as high-quality genomes. GTDB-tk ver. 1.5.1 software (r202) was used for the taxonomic classification of bins (Chaumeil *et al.*, 2020). High-quality *Myxococcota* metagenomes from E1 were obtained in our previous studies (Kuroda *et al.*, 2023; Tomita *et al.*, 2023). Six activated sludge E1 samples were used for DNA extraction and shotgun metagenomic sequence ana­lyses. The 6 metagenomic sequences were co-assembled to obtain long and high-quality contigs.

A genome tree was constructed using the concatenated phylogenetic marker genes of the obtained bin and the phylum *Myxococcota*. Conserved marker genes were identified using “gtdbtk identify” with default parameters and aligned to reference genomes using “gtdbtk align” with taxonomic filters (-taxa_filter p__Myxococcota, p__Myxococcota_A, p__Myxococcota_B) (Chaumeil *et al.*, 2020). A phylogenetic tree was constructed using IQ-TREE version 2.1.4-beta (-B 1000) with an automatically optimized substitution model (LG+F+R10) (Minh *et al.*, 2020).

### Estimation of secondary metabolite synthetic genes of myxobacteria in activated sludge

All bins belonging to the phylum *Myxococcota* were annotated through the combination of Prokka v1.14.6 (Seemann, 2014), DRAM software (Shaffer *et al.*, 2020), and GhostKOALA (Kanehisa *et al.*, 2016) with manual annotation. To predict secondary metabolite gene clusters, we used antiSMASH (version 5.0) (Blin *et al.*, 2019). In addition, Natural Product Domain Seeker (NaPDoS) (Ziemert *et al.*, 2012) was employed to identify the C domains in non-ribosomal peptide synthase (NRPS) and KS domains in polyketide synthase (PKS) in *Myxococcota* bins with default parameters. The C and KS domains were subjected to a phylogenetic ana­lysis using NaPDoS to generate phylogenetic trees. To correctly estimate the phylogenetic positions of KS domain sequences, we excluded genes involved in the synthesis of fatty acids and polyunsaturated fatty acids. To assess social behavior and cell contact-dependent prey killing in *Myxococcota* bins obtained from activated sludge samples, a BLASTP-based homology search (e-value 1e-5, qcovs >50%) was performed against the *M. xanthus* DK1622 genome (GCA_000012685.1). Principal component ana­lyses (PCA) based on glycoside hydrolases (GHs) and peptidases were conducted using PAST4 software (Hammer *et al.*, 2001). Bins with Calvin–Benson–Bassham (CBB) cycles were selected and annotated with photosynthetic gene clusters using the default parameters from eggNOG (Cantalapiedra *et al.*, 2021) and KEGG software. Genes involved in phosphate accumulation in polyphosphate-accumulating bacteria were referenced, and the presence or absence of polyphosphate accumulation was assessed using a BLASTP-based homology search (e-value <1e-5, qcovs >50%) and GhostKOALA.

### Deposition of DNA sequence data

Raw sequence data from activated sludge samples F1, G1, and H1 have been deposited in the DDBJ Sequence Read Archive (SRA) database (DRA018446). Sequence data from activated sludge sample E1 were obtained from the DDBJ SRA database (DRA015582 and DRA016086) deposited in a previous study (Kuroda *et al.*, 2023). *Myxococcota* bin data from this study are available at figshare: https://doi.org/10.6084/m9.figshare.25702542.

## Results and Discussion

### Detection of Myxococcota recovered from activated sludge systems through 16S rRNA gene and metagenomic sequence ana­lyses

**Fig. 1.**
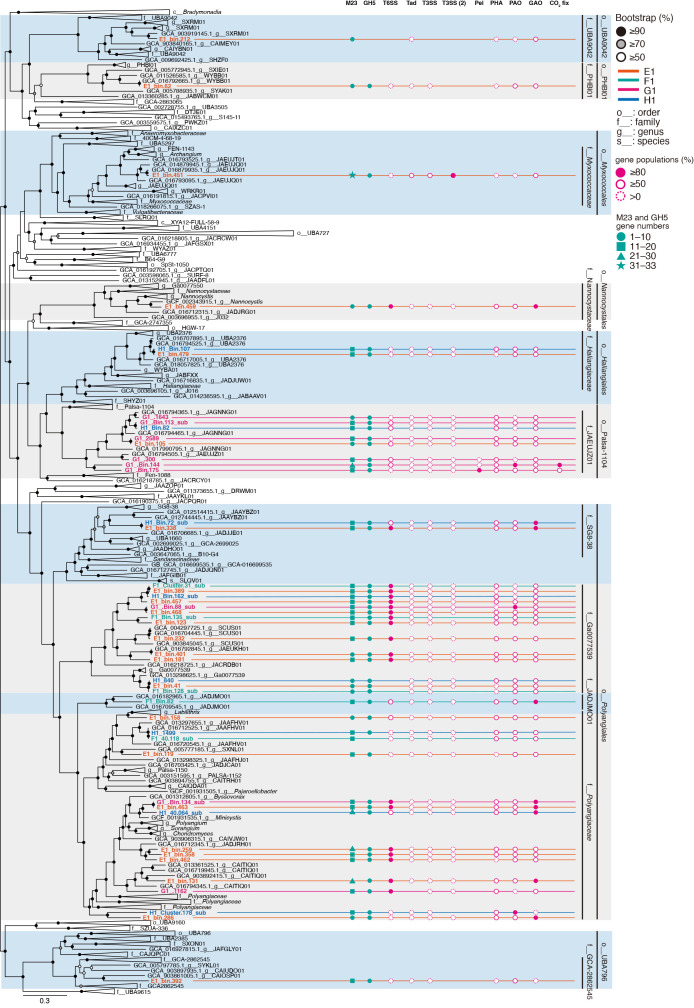
Genome tree of the phylum *Myxococcota* based on concatenated phylogenetic marker genes in GTDBtk 2.0.0 (ver. 207). Black, gray, and white circles indicate ultrafast bootstrap (1,000 replicates)-supported probabilities at >90%, >70%, and >50%, respectively. Magenta circles, solid lines with white circles, and dashed white circles show gene populations of ≥80%, ≥50%, and >0%, respectively. Green circles, squares, triangles, and stars indicate the detected numbers of M23 and GH5 families ranging from 1–10, 11–20, 21–30, and 31–33, respectively. M23 and GH5: M23 and GH5 families, T6SS: Type VI secretion systems, Tad: tight adherence (Tad)-like systems, T3SS: Type VI secretion systems, T3SS(2): Type VI secretion systems (2), Pel: Pel genes, PHA: polyhydroxyalkanoate production, PAO and GAO: polyphosphate- and glycogen-accumulating organism-related metabolism, and CO_2_ fix: CO_2_ fixation pathways with ribulose 1,5-bisphosphate carboxylase/oxygenase (RuBisCO), the Calvin–Benson–Bassham (CBB) cycle, carotenoid metabolism, and bacteriochlo­rophyll production.

Microbiome ana­lyses based on the 16S rRNA gene and shotgun metagenomic sequencing were performed to assess the ecology of myxobacteria in the activated sludge systems from four WWTPs (E1, F1, G1, and H1). The phylum *Myxococcota* was present in activated sludge at an abundance of between 3.6 and 9.5% of all prokaryotes (Table S1). Through metagenomic sequencing, 46 metagenomic bins belonging to the phylum *Myxococcota* (average completeness: 90.5%; average contamination: 5.06%) of the 340‍ ‍bins (average completeness: 91.6%; average contamination: 3.40%) were recovered. These 46 bins were classified to the orders *Polyangiales* (31 bins), Palsa-1104 (8 bins), *Haliangiales* (2 bins), *Nannocystales* (1 bin), *Myxococcales* (1 bin), UBA796 (1 bin), PHBI796 (1 bin), PHBI01 (1 bin), and UBA9042 (1 bin) based on GTDB-based phylogenies (Chaumeil *et al.*, 2020) (Fig. 1, Table 1 and S2). Among the 46 *Myxococcota* bins, only 18 and 4 belonged to known family and genus clades (genera *Nannocystis* and *Minicystis*), respectively, indicating that most of the detected *Myxococcota* were placed in uncultured clades at the family or genus level. The recovered *Myxococcota* bins showed larger genome sizes, ranging between 6.6 and 10.7‍ ‍Mb (average 9.2±1.8‍ ‍Mb), than general bacterial genomes, such as *E. coli* (4.5–5.5‍ ‍Mb), and the genome sizes of myxobacteria were similar to those obtained in a previous study (Wang *et al.*, 2023). Based on the 16S rRNA gene-based myxobacterial composition, the class *Polyangia* was the dominant microbe in the phylum *Myxococcota* at the 4 WWTPs (Table S1). In addition, 42 out of 46 metagenomic bins belonged to the class *Polyangia* (Table S1 and S2). Therefore, the metagenomic bins of the predominant *Myxococcota* appeared to have been correctly recovered from activated sludge samples in the present study.

### Predatory functions

**Fig. 2.**
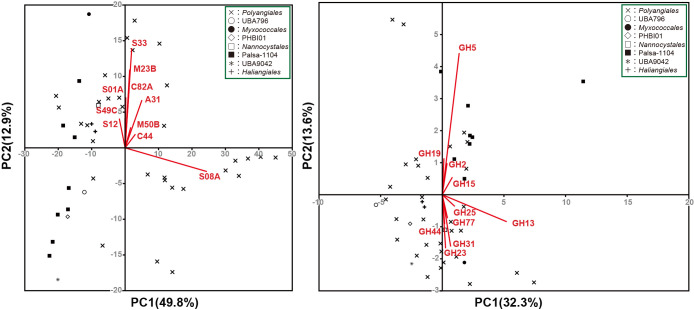
Principal component ana­lysis (PCA) of the number of genes for (A) peptidase and (B) glycoside hydrolase. Each number with a letter indicates a family of peptidases or glycoside hydrolases.

Typical myxobacteria, such as *M. xanthus*, predate other bacteria (*e.g.*, *E. coli* and *Saccharomyces cerevisiae*) by producing degradative enzymes (Thiery *et al.*, 2022). To evaluate their predatory potential, principal component ana­lysis (PCA) was performed using the number of identified peptidases and glycoside hydrolases in *Myxococcota* bins (Fig. 2, Table 1, S3, and S4). Some bins belonging to the order *Polyangiales* were placed together in the same direction of the biplot of M23B family peptidases (Fig. 2A and Table S4), indicating that some bins belonging to the order *Polyangiales* possessed many M23 family genes. The M23 family includes peptidase enzymes that cleave peptide bonds, such as the peptidoglycan of bacterial cell walls (Razew *et al.*, 2022) (Fig. 2A). Furthermore, bacteriocins and autolysins, which belong to the M23 family, exert a number of effects, such as the inhibition of protein synthesis and antibacterial activity against other bacteria (Cleveland *et al.*, 2001). Regarding glycoside hydrolases, many genes of the GH5 family, which contain cellulases and exoglucanases, were identified in the Palsa-1104 and *Polyangiales* bins (Fig. 1 and 2B). We previously revealed that *Myxococcota* bacteria were predominant in activated sludge systems in which wastewater treatment was stable (Kuroda *et al.*, 2023). In addition, *Myxococcota* have been identified as active predators in activated sludge processes (Zhang *et al.*, 2023). These findings imply that *Myxococcota* prey on several operational problem-causing bacteria, such as bulking-causing filamentous bacteria, to maintain a stable environment for wastewater treatment. Some myxobacteria were shown to be capable of producing β–barrel-glucanase (GluM), which allowed them to prey on fungi with β-1,6-glucane in the cell wall (Li *et al.*, 2019). Therefore, myxobacteria belonging to Palsa-1104 and *Polyangiales* obtained in this study may prey on fungi in addition to bacteria in activated sludge. Further investigations involving physiological and enzymatic experiments are needed to clarify the predatory mechanisms of *Myxococcota* in activated sludge.

**Fig. 3.**
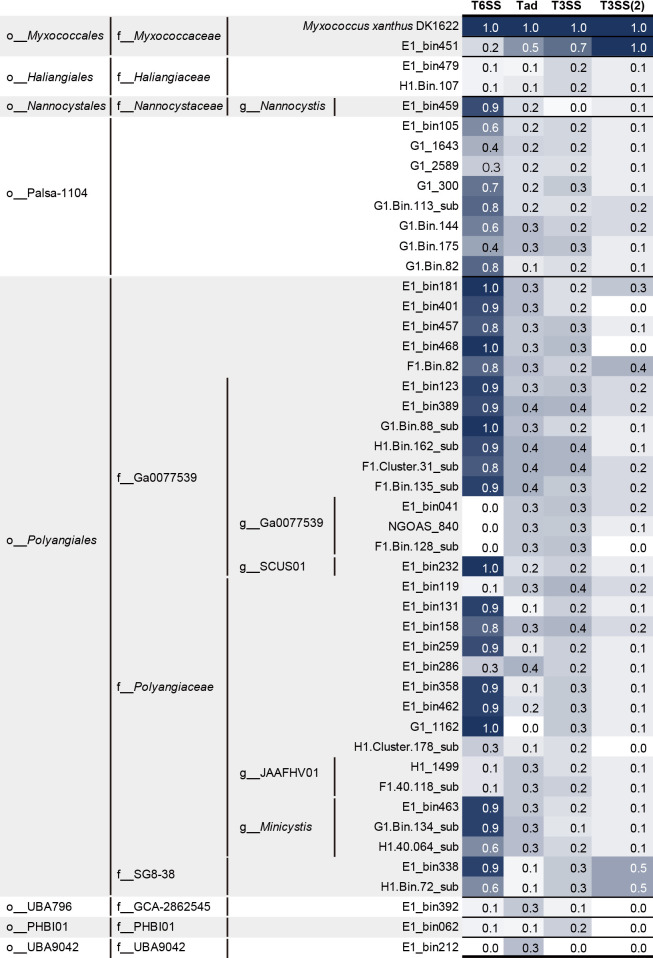
Possible cell contact-dependent predatory functions of metagenomic bins of the phylum *Myxococcota* in activated sludge systems. Type VI secretion systems (T6SS), tight adherence (Tad)-like systems, and Type III secretion systems (T3SS) were annotated with the genome of *Myxococcus xanthus* DK1622 (GCA_000012685.1) at thresholds of ≥25% amino acid identity, ≤1e-5 e-value, and ≥50% query coverage per subject. The number of each secretion system indicates gene proportions in the metagenomic bin. Gene populations were calculated based on the number of genes in each family. Detailed annotation results are listed in Table S5.

In addition, *M. xanthus* DK1622 possesses cell contact-dependent predatory functions that digest bacterial cells to obtain nutrients (Seef *et al.*, 2021). These predatory functions require several secretion systems, such as T2SS, T3SS, T4SS, and T6SS (Thiery *et al.*, 2022), and *M. xanthus* DK1622 has been predicted to utilize two types of T3SS and the “Kill complex” of Tad-like secretion for the export of several digestive enzymes from cells (Seef *et al.*, 2021; Thiery *et al.*, 2022). To estimate the potential functions of the bins in this study, an amino acid-based homology search with the CDSs of *M. xanthus* DK1622 was performed with the threshold of 1e-5 ≥e-value (Fig. 1 and 3, Table S5). The bin in the family *Myxococcaceae* possessed ≥50% of the homologs of Tad-like secretion, T3SS, and T3SS (2) (Fig. 3), which was consistent with our previous findings showing that only *Myxococcaceae* bins had widely conserved Tad-like secretion systems in the genomes (Kuroda *et al.*, 2023). Furthermore, bins belonging to *Nannocystis*, Palsa-1104, *Polyangiales*, Ga0077539, SCUS01, *Polyangiaceae*, *Minicystis*, and SG8-3 encoded T6SS. Mutations in *M. xanthus* T6SS did not affect the cell contact-dependent predation of *E. coli* (Seef *et al.*, 2021); therefore, these taxa may not have cell contact-dependent predatory functions or may have different predatory mechanisms from those of the family *Myxococcaceae*.

### Social behavior

In the present study, the presence of gene clusters related to motility and fruiting body formation was investigated by amino acid sequence-based homology searches using *M. xanthus* DK1622 as the reference genome (Table 1, S2, and S6). *Myxococcota* genomes in activated sludge systems shared (>0%) EBP, MrpC, Nla24, EPS production, A-signal, aggregation-sporulation-fruiting body formation, developmental timers, adventurous motility, social motility, OME, and chemosensory pathways/rippling modules. *M. xanthus* moves on solid surfaces using type IV pili, which have independent flagellar motility and frequently come into contact with other myxobacterial cells (Chen and Nan, 2022). TraA and TraB on the outer membrane act as cell surface receptors that recognize whether a neighboring cell is clonal (Vassallo *et al.*, 2015). OME exchanges outer membrane proteins, lipids, and toxic compounds across the coupled outer membrane. When myxobacteria, which have different types of toxins depending on their phylogeny, exchange proteins with non-self cells, each cell recognizes different myxobacteria through immunity proteins (Sah and Wall, 2020). The combination of TraA and OME recognition allows clonal cells to aggregate and form social communities. Since homologs of TraA and TraB have been identified in *Myxococcota* genomes, they use a clonal cell-recognition mechanism similar to that of *M. xanthus*. Pili are important proteins that regulate S motility. Through the annotation of pili genes relevant to S motility, only the homolog of pilA was found in bins belonging to Palsa-1104 and *Polyangiales*. PilA synthesizes pili and drives cells in a forward-pulling motion. However, in other myxobacteria, such as *M. xanthus* (Sharma *et al.*, 2018), pilB and pilT performed the same functions as pilA. In addition, this study identified pilB and pilT in several bins of *Myxococcota* that have no pilA. Therefore, other genes, such as pilB and pilT, may complement the role of pilA in S motility (Table S6). In contrast, some FruA and C-signal modules were only detected in bins belonging to Palsa-1104 and *Polyangiales*. The FruA module is relevant for controlling aggregation associated with fruiting body formation and sporulation (Campbell *et al.*, 2015), and the C-signal module contains the PopC, PopD, and CsgA genes, which control the onset of fruiting body formation (Boynton and Shimkets, 2015). The CsgA gene is a precursor of the C-signal, which is necessary for fruiting body formation and sporulation, and promotes sporulation outside the fruiting body by excessive secretion. In addition, PopC protease hydrolyzes CsgA to produce C-signals. PopD inhibits PopC protease activity by forming a complex with the PopC C-signal, which recognizes neighboring myxobacterial cells and activates signaling (ActA, ActB, and FruA) after secretion outside the outer membrane. In the C-signal module, only homologs of PopC were identified in the genomes of the Palsa-1104 and *Polyangiales* bins (Table S6). Therefore, Palsa-1104 and *Polyangiales* in this study may not have been able to form fruiting bodies due to the lack of CsgA genes. To the best of our knowledge, fruiting body formation in the orders Palsa-1104 and *Polyangiales* was not detected in activated sludge, suggesting that these taxa do not require fruiting body formation in their habitats. Furthermore, we did not identify the complete set of genes relevant to fruiting body formation from the 46 activated sludge *Myxococcota* bins, including the family *Myxococcaceae* (Table 1, S2, and S6). This may be explained by the presence of rich nutrients in wastewater because this social behavior is triggered by the starvation of myxobacteria in the environment; therefore, fruiting body formation is not required for adaptation to activated sludge environments. Further investigations are needed to clarify whether the lack of essential genes for fruiting body formation and sporulation is due to genomic incompleteness by reconstructing the complete genome.

### Metabolic potential relevant to organic carbon, nitrogen, and sulfur utilization

**Fig. 4.**
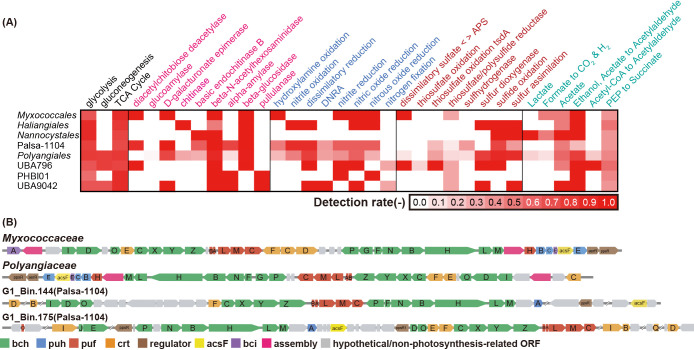
Metabolic potentials of metagenomic bins of the phylum *Myxococcota* in activated sludge systems. (A) Heatmap showing the metabolic functions of *Myxococcota* in activated sludge at the order level. (B) Potential phototrophic *Myxococcota* (G1_Bin.144 and G1_Bin.175) in activated sludge systems and photosynthetic gene clusters (PGCs) compared with known PGCs in a previous study (Li *et al.*, 2023). Genes for bacteriochlo­rophyll biosynthesis, reaction center proteins, reaction center assembly proteins, and carotenoid biosynthesis genes are denoted as bch (green)/bci (purple), puf(red), puh (blue), and crt (orange), respectively. Hypothetical genes are shown in gray. Details of the annotation are provided in Table S10.

To assess metabolic information on the phylum *Myxococcota* in activated sludge environments, the potential metabolism of carbon, nitrogen, and sulfur was annotated using the GhostKOALA annotation tool (Fig. 4A and Table S7). Most *Myxococcota* bins had almost complete meta­bolic‍ ‍pathways for glycolysis and the TCA cycle. Bins belonging to *Myxococcales*, *Haliangiales*, *Nannocystales*, Palsa-1104, *Polyangiales*, and UBA796 encoded β-N-acetylhexosaminidase in these genomes. This enzyme has been reported to play a role in chitin degradation and the formation of chitinase derivatives in marine chitin-degrading bacteria (Keyhani and Roseman, 1996). In addition, some bins in the orders *Polyangiales* and Palsa-1104 possess beta-glucosidase, which is relevant for the degradation of cellulose. *Bacteria* in the genus *Sorangium* belonging to the family *Polyangiaceae* have been identified as cellulose-utilizing myxobacteria (Schneiker *et al.*, 2007). This finding suggests that bins in G1_Bin.134_sub, E1_bin.463, and H1_40.064_sub have cellulose availability because of their close phylogenetic placement with the genus *Sorangium*. In potential nitrogen metabolism, bins in *Myxococcales*, *Haliangiales*, *Polyangiales*, and Palsa-1104 have the genes nirK/nirS, NorB/C, and NosZ, suggesting that *Myxococcota* bins contribute to denitrification in activated sludge systems. Regarding sulfur utilization, genes involved in sulfide oxidation and sulfur assimilation are shared in the phylum *Myxococcota*, except for *Myxococcales* and PHBI01. Sulfide dioxygenases (SDOs), sulfide-quinone oxidoreductase (sqr), the flavoprotein of sulfide dehydrogenase (fccB), and sulfite reductase (ferredoxin; sir)) were annotated. Therefore, *Myxococcota*, except for *Myxococcales* and PHBI01, may catalyze the oxidation of S^0^ to sulfite, detoxify hydrogen sulfide, and reduce sulfite (Wu *et al.*, 2017).

### Biofilm formation, PHA production, and phosphorus/glycogen accumulation

*M. xanthus* secretes extracellular polysaccharides during its migration and colonization (Zhou and Nan, 2017). A recent study identified pel genes in the MAG of *Myxococcota* in activated sludge systems (Dueholm *et al.*, 2023). Pel is widely conserved in Gram-positive and -negative bacteria, contributes to biofilm and pellicle formation, and mediates cell-cell interactions (Jennings *et al.*, 2015). In the present study, pel genes were identified in three bins (G1_300, G1_Bin.144, and G1_Bin.175) belonging to Palsa-1104. Conversely, no signal peptides were observed in the Pel genes in this study, suggesting that Pel polysaccharides were not secreted extracellularly into activated sludge and also that Pel did not contribute to biofilm formation in activated sludge (Fig. 1, Table S7, S8, and S9). Genes relevant to the production of polyhydroxyalkanoate (PHA) were newly found in most *Myxococcota* genomes (Table S8 and S9). The accumulation of PHA plays an important role in stress tolerance and biofilm formation by *Pseudomonas* spp. (Pham *et al.*, 2004). Therefore, the intracellular production of PHA from *Myxococcota* in activated sludge may control biofilm formation for adaptation to complex environmental conditions.

In activated sludge systems, polyphosphate- and glycogen-accumulating organisms (PAOs and GAOs) are important bacteria that control biological phosphorus removal processes. These microorganisms have been found in the bacterial flocs of phosphorus removal wastewater treatment processes (Kodera *et al.*, 2013), and PAO-GAO competition often results in the deterioration of the phosphorus removal process. Therefore, controlling the abundance of these microorganisms is essential for the effective removal of phosphorus from wastewater. PAOs (such as *Ca.* Accumulibacter phosphatis) and GAOs (including *Ca.* Defluviicoccus tetraformis strain TFO71) have similar PHA metabolism (glgABCEX) and glycogen metabolism (phaABCEJ and fabDFG) genes (amino acid sequence similarity: up to 72%) when coexisting under anaerobic-aerobic conditions, which may be due to similar genetic adaptations as a result of selection pressure for survival in an enhanced biological phosphorus removal (EBPR) bioreactor (Nobu *et al.*, 2014). Comparisons of *Myxococcota* in the present study with the genomic characteristics of PAOs and GAOs showed no significant differences in the presence or absence of genes involved in PHA synthesis/degradation and glycogen metabolism (Fig. 1 and Table S8). Therefore, PHA and glycogen metabolism by *Myxococcota* bacteria may function as PAOs and/or GAOs to store organic matter anaerobically and obtain energy from stored PHA (Acevedo *et al.*, 2012). In previous studies, positive correlations were observed between mle1-27, another *Myxococcota*, and organic matter removal rates (Zhang *et al.*, 2023). In addition, mle1-27 positively correlated with the genus *Tetrasphaera*, which is known as a PAO. To further elucidate the contribution of *Myxococcota* in activated sludge to phosphorus removal during wastewater treatment, physiological and microscopic experiments are required.

### Carbon fixation

A recent metagenomic ana­lysis revealed that the families *Houyibacteriaceae*, *Myxococcaceae*, *Nannocystaceae*, *Polyangiaceae*, *Kuafubacteriaceae*, and *Sandaracinaceae* in the phylum *Myxococcota* have metabolic pathways that are important for photosynthesis, including RuBisCO, the CBB cycle, carotenoid metabolism, and bacteriochlo­rophyll production (Li *et al.*, 2023). *M. xanthus* is a heterotrophic bacterium with glycolytic and TCA cycles; however, this study reported some Candidatus species potentially capable of autotrophic metabolism. In this study, G1_Bin.144 and G1_Bin.175, belonging to Palsa-1104 and recovered from G1, possessed RuBisCO and CBB cycles, suggesting that these bins fix CO_2_ through the carbonate fixation pathway (Fig. 1 and Table S10).

A BLASTP homology search of the NCBI database identified RuBisCO in these bins as type I RuBisCO. Type I RuBisCO catalyzes carbon fixation reactions during photosynthesis and is widely conserved in plants and photosynthetic bacteria (Liu *et al.*, 2023). In addition, biosynthetic pathways for carotenoids and chlo­rophyll were identified, supporting the potential for autotrophic metabolism in the *Myxococcota* bins (Fig. 4B, Table S7 and S10). Based on the annotations of photosynthetic pathways, G1_Bin175 and G1_Bin144 had carotenoid and bacteriochlo­rophyll synthesis pathways. Previous studies reported that the photosynthetic gene clusters of *Myxococcota* contained diverse carotenoid biosynthetic pathways, and carotenoids may function as light harvesting accessory pigments and in photoprotection against free radicals or as antimicrobial agents and compounds with alternative functions (Li *et al.*, 2023). In the present study, G1_Bin.144 had several metabolic pathways capable of catalyzing the precursor bacteriochlorophylide and producing bacteriochlo­rophyll. G1_Bin.175 also lacked a part of the bacteriochlo­rophyll biosynthesis pathway; however, a gene that catalyzes bacteriochlo­rophyll production was identified and may produce bacteriochlo­rophyll. These results revealed that unique *Myxococcota* Palsa-1104 may have both heterotrophic and autotrophic metabolic pathways, providing additional insights into the phototrophic potential of *Myxococcota*.

### Prediction of secondary metabolism

A total of 533 BGCs were identified based on the evaluation of secondary metabolite biosynthesis genes using antiSMASH (Table S11). *Nannocystales* (26 BGCs per bin), UBA796 (19), Palsa-1104 (16), and *Polyangiales* (11) had higher numbers of BGCs per genome than the other orders of *Myxococcota*. In addition, BGCs classified as NRPS and PKS were mainly encoded by Palsa-1104 (50 BGCs) and *Haliangiales* (88 BGCs). Bacteriocins and aryl polyenes were frequently found in annotated BGCs. Bacteriocins are antibiotics that are conserved in a wide range of bacteria and act on closely related species (Cleveland *et al.*, 2001). Myxobacteria exchange toxins as a recognition mechanism to avoid the grouping of different species during OME (Vassallo *et al.*, 2015). Therefore, bacteriocins are highly conserved among myxobacterial cells to identify themselves. Aryl polyenes, which are structurally similar to carotenoids, have also been reported to protect cells from damage caused by reactive oxygen species and promote biofilm formation by EPS in *E. coli*. EPS production by *M. xanthus* is important for smooth S cell movement and colony formation (Johnston *et al.*, 2021). These findings suggest that *Myxococcota* in activated sludge use bacteriocins during OME to avoid grouping with different species and promote the production of EPS. Since the production of EPS in *M. xanthus* is essential for aggregation, fruiting body formation, and gliding, aryl polyenes may be involved in biofilm or polysaccharide production in *Myxococcota*. Secondary metabolic biosynthetic genes capable of producing aryl polyenes and bacteriocins were detected in some *Polyangiales* and Palsa-1104 strains in the present study, indicating that these substances are produced and used during OME and cell growth.

**Fig. 5.**
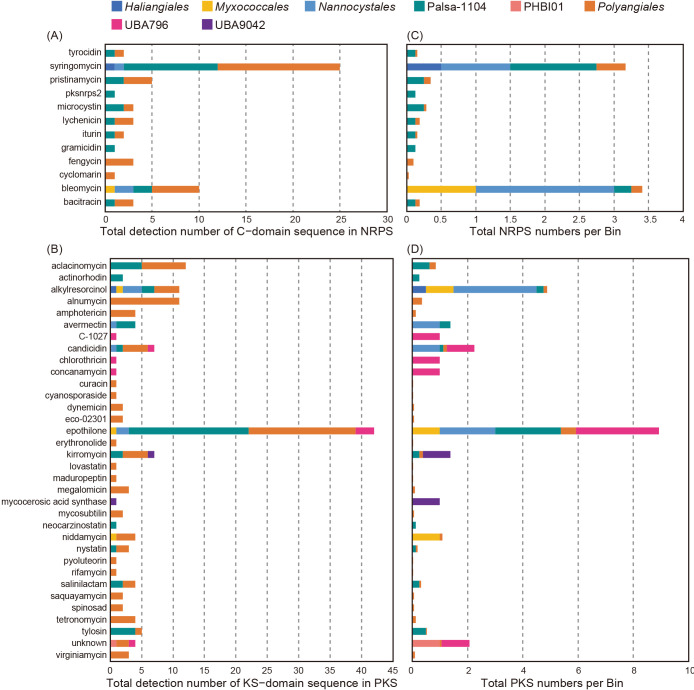
Results on the identification of secondary metabolite genes (C-domain sequence for non-ribosomal peptide synthase [NRPS] and KS-domain sequence for polyketide synthase [PKS]) in *Myxococcota* metagenome-assembled genomes using NaPDoS software. (A) and (B) Total numbers of NRPS and PKS, respectively. (C) and (D) Numbers of NRPS per bin and PKS per bin, respectively.

Furthermore, we identified the C domain of NRPS and KS domain of PKS as the secondary metabolite biosynthetic genes of *Myxococcota* in activated sludge using NaPDoS software based on an amino acid sequence homology search of the NaPDoS database (KS and C domains) (Fig. 5, Table S12 and S13) (Ziemert *et al.*, 2012). In NRPS, 66 amino acid sequences of the C domain annotated as bacitracin, bleomycin, cyclomarin, fengycin, gramicidin, iturin, microcystin, pksnrps2, pristinamycin, syringomycin, and tyrocidin were detected in *Myxococcota* genomes. Fengycin and iturin may be useful biological pesticides in agricultural systems (Fira *et al.*, 2018). In PKS, we detected 373 KS domain amino acid sequences; among these, 228 sequences were classified as being involved in the synthesis of fatty acids and polyunsaturated fatty acids. The remaining 145 amino acid sequences belong to virginiamycin, tylosin, tetronomycin, salinilactam, rifamycin, pyoluteorin, polyunsaturated fatty acids, nystatin, nidamycin, neocarzinostatin, mycocerosic acid synthase, megalomicin, lovastatin, and kirromycin. Virginiamycin and rifamycin in the KS domain have also been used as antibacterial agents (Cocito, 1979; Batiha *et al.*, 2020). In contrast, NRPS and PKS in this study were homologous to known amino acid sequences ranging from 21–47% (e-value: 7.0e-6 to 1.0e-100) and 23–73% (2.0e-6 to 1.0e-172), respectively, with most sequences having low homology. Therefore, *Myxococcota* in activated sludge in the present study may have novel secondary metabolite biosynthesis genes. A previous study reported that *M. xanthus* secreted secondary metabolites that may be used to kill and degrade other bacterial cells (Thiery and Kaimer, 2020). Therefore, these novel secondary metabolites may be used to prey on other bacteria in the activated sludge process. To prove the mechanisms, the isolation/cultivation of the predominant *Myxococcota* microorganisms and *in vitro* tests on predation activities are required.

## Conclusion

To elucidate the potential metabolic characteristics of myxobacteria in activated sludge systems, we evaluated 46 metagenomic bins of the phylum *Myxococcota* recovered from four WWTPs. Through metagenomic ana­lyses, we discovered novel potential functions for *Myxococcota* in activated sludge as follows: 1) Palsa-1104 and Polyangiales bins have the potential for cell wall degradation due to the presence of many genes in the families M23 and GH13, 2) cell contact-dependent predatory functions were conserved in the family *Myxococcaceae*, 3) fruiting body formation-related genes were lacking in the *Myxococcota* genomes, 4) two bins belonging to Palsa-1104 had phototrophic gene clusters with heterotopic metabolism, and 5) several novel secondary metabolite biosynthesis gene clusters were present in the *Myxococcota* genomes. These results indicate that the diverse metabolic functions of *Myxococcota* are hidden in activated sludge systems. Further investigations are needed to clarify whether the absence of known core genes for cell contact-dependent predatory functions and fruiting body formation is due to genomic incompleteness or the presence of novel gene clusters. In addition, metatranscriptomics is necessary to prove these conclusions and clarify the mechanisms underlying the ecophysiology of *Myxococcota* microorganisms in activated sludge systems.

## Citation

Kurashita, H., Hatamoto, M., Tomita, S., Yamaguchi, T., Narihiro, T., and Kuroda, K. (2024) Comprehensive Insights into Potential Metabolic Functions of *Myxococcota* in Activated Sludge Systems. *Microbes Environ ***39**: ME24068.

https://doi.org/10.1264/jsme2.ME24068

## Supplementary Material

Supplementary Material 1

Supplementary Material 2

## Figures and Tables

**Table 1.** Genes relevant to the social behavior of *Myxococcota* metagenome-assembled genomes obtained from activated sludge systems.

Classification	Genomic features					Average detection proportions of social behavior-related gene modules^g^												
	comp^a^ (%)	cont^b^ (%)	cov^c^ (X)	size^d^ (Mb)		A	B	C	D	E	F	G	H	I	J	K	L	M
*M. xanthus* DK1622^e^	98.7	2.0	—	9.1		1.0	1.0	1.0	1.0	1.0	1.0	1.0	1.0	1.0	1.0	1.0	1.0	1.0
*Myxococcales* (1)^f^	94.1	3.7	73	9.7		0.8	0.8	1.0	1.0	1.0	0.0	0.8	0.4	0.8	1.0	0.9	1.0	0.9
*Haliangiales* (2)	92.9	3.4	28	8.8		1.0	0.9	1.0	0.4	0.0	0.0	0.6	0.3	0.5	0.8	0.7	1.0	0.7
*Nannocystales* (1)	97.1	6.3	103	10.7		0.9	0.8	1.0	0.4	1.0	0.0	0.8	0.3	0.8	0.7	0.6	1.0	0.7
Palsa-1104 (8)	90.6	5.4	58	8.3		0.9	0.7	1.0	0.5	0.9	0.3	0.7	0.4	0.4	0.7	0.6	1.0	0.7
*Polyangiales* (31)	90.1	5.4	39	9.6		0.9	0.8	1.0	0.6	0.6	0.1	0.6	0.4	0.7	0.6	0.7	1.0	0.6
UBA796 (1)	90.4	4.0	39	8.9		0.7	0.4	0.5	0.3	0.0	0.0	0.4	0.3	0.3	0.7	0.6	0.5	0.2
PHBI01 (1)	89.5	1.3	33	7.4		0.7	0.8	1.0	0.4	0.0	0.0	0.3	0.3	0.3	0.6	0.4	1.0	0.2
UBA9042 (1)	85.5	2.0	20	6.6		0.7	0.4	0.5	0.5	0.0	0.0	0.5	0.3	0.5	0.5	0.6	1.0	0.3

a: average completeness b: average contamination c: average coverage e: reference genome (GCA_000012685.1) d: average genome size f: number of bins g: Proportions of social behavior-related gene modules of the phylum *Myxococcota* calculated based on the reference genomes of *M. xanthus*. A: EBP module B: MrpC module C: Nla24 module D: EPS production E: FruA module F: C-signal G: A-signal H: Aggregation, sporulation, and fruiting body formation I: Development timers J: Adventurous (A) motility K: Social (S) motility L: Outer Membrane Exchange (OME) M: Chemosensory pathways/Rippling
