# Supplementary material for: Comprehensive Insights into Potential Metabolic Functions of Myxococcota in Activated Sludge Systems
Source: Microbes Environ. 2024 Dec 27;39(4):ME24068. doi: 10.1264/jsme2.ME24068 (PMC11821767; doi:10.1264/jsme2.ME24068)
Supplement: Supplementary file 1 — Supplementary Material 1 [file 39_24068_s1.docx]

**Legends of supplementary tables**

Table S1. Abundance of 16S rRNA gene sequences at phylum and order levels in the activated sludge systems from four wastewater treatment plants.

Table S2. Summary of the *Myxococcota* metagenomic bins obtained in this study.

Table S3. Number of glycoside hydrolase related genes from *Myxococcota* in activated sludge.

Table S4. Number of peptidase related genes from *Myxococcota* in activated sludge.

Table S5. Locus tags for possible cell contact-dependent predatory functions of *Myxococcota* in this study.

Table S6. Locus tags for genes encoding social behavior of the myxobacteria in this study.

Table S7. Summary of the metabolic functions of *Myxococcota* in this study.

Table S8. Locus tags for genes encoding PHA synthesis/degradation, polyphosphate/glycogen accumulation, and Pel synthesis in this study.

Table S9. Presence (1)/absence (0) of genes encoding PHA synthesis/degradation, polyphosphate/glycogen accumulation, and Pel synthesis in this study.

Table S10. Locus tags for genes relevant to photosynthetic gene clusters in this study.

Table S11. Summary of identified secondary metabolite biosynthesis genes using antiSMASH software.

Table S12. Summary of the identified C domain in non-ribosomal peptide synthase based on NaPDoS software.

Table S13. Summary of the identified KS domain in Polyketide synthase based on NaPDoS software.
